# PD-L1 is highly expressed in Enzalutamide resistant prostate cancer

**DOI:** 10.18632/oncotarget.2703

**Published:** 2014-11-06

**Authors:** Jennifer L. Bishop, Alexander Sio, Arkhjamil Angeles, Morgan E. Roberts, Arun A. Azad, Kim N. Chi, Amina Zoubeidi

**Affiliations:** ^1^ Vancouver Prostate Centre, Vancouver, BC, Canada; ^2^ Department of Microbiology and Immunology, University of British Columbia, Vancouver, BC, Canada; ^3^ Department of Medicine, Division of Medical Oncology, BC Cancer Agency, University of British Columbia, Vancouver, BC, Canada; ^4^ Department of Urologic Sciences, University of British Columbia, Vancouver, BC, Canada

**Keywords:** Enzalutamide resistant CRPC, Immunotherapy, PD-L1

## Abstract

Efficacy of Enzalutamide (ENZ) in castration resistant prostate cancer (CRPC) patients is short-lived. Immunotherapy like T cell checkpoint blockade may improve patient survival. However, when and where checkpoint molecules are expressed in CRPC and whether immune evasion is a mechanism of ENZ resistance remains unclear. Thus, we investigated whether clinically relevant immunotherapy targets, specifically PD-L1/2, PD-1 and CTLA-4, are upregulated in ENZ resistant (ENZR) patients and in a pre-clinical model of ENZ resistance. We show for the first time that patients progressing on ENZ had significantly increased PD-L1/2^+^ dendritic cells (DC) in blood compared to those naïve or responding to treatment, and a high frequency of PD-1^+^T cells. These data supported our pre-clinical results, in which we found significantly increased circulating PD-L1/2^+^ DCs in mice bearing ENZR tumors compared to CRPC, and ENZR tumors expressed significantly increased levels of tumor-intrinsic PD-L1. Importantly, the expression of PD-L1 on ENZR cells, or the ability to modulate PD-L1/2^+^ DC frequency, was unique to ENZR cell lines and xenografts that did not show classical activation of the androgen receptor. Overall, our results suggest that ENZ resistance is associated with the strong expression of anti-PD-1 therapy targets in circulating immune cells both in patients and in a pre-clinical model that is non-AR driven. Further evaluation of the contribution of tumor vs. immune cell PD-L1 expression in progression of CRPC to anti-androgen resistance and the utility of monitoring circulating cell PD-L1 pathway activity in CRPC patients to predict responsiveness to checkpoint immunotherapy, is warranted.

## INTRODUCTION

Therapies targeting tumor-fueling androgens have been mainstay treatments of advanced prostate cancer (PCa) for almost 5 decades. However, the inevitable recurrence of tumors after anti-androgen treatment leads to incurable castration-resistant prostate cancer (CRPC). Recently, a number of new drugs have been approved that prolong survival in CRPC patients, including the potent anti-androgen Enzalutamide (ENZ). However, treatment benefits of ENZ are short-lived, and progression on ENZ is inevitable [1], a phenomenon that can be modelled *in vitro* and *in vivo* [2,3].

While continued dependence on androgen receptor (AR) signalling in CRPC creates demand for novel androgen targeted therapies, immunotherapies may provide a complimentary avenue to improve survival in men with CRPC, especially in patients resistant to hormone therapy [4]. Indeed, anti-androgen treatment may abrogate the tolerogenic effect CRPC can have on local and systemic immune responses [5]. Thus, intervention with immunotherapy may be most amenable in patients that have received anti-androgens, however, selection and sequencing of effective immunotherapies for CRPC remains unclear. This is underscored by the discordant clinical responses observed in trials of CRPC patients receiving the checkpoint blockade immunotherapies Ipilimumab vs. anti-PD-1 antibodies, which prevent CTLA-4 and PD-1 mediated T cell suppression, respectively. For example, whereas Ipilimumab induced >50% PSA decline in 8 out of 50 men with metastatic CRPC [6], anti-PD1 treatment failed to produce an objective response in a separate small trial of 17 CRPC patients [7]. These data and the strong correlation between tumor expression of the PD-1 ligand, PD-L1, and positive responses to PD-1 blockade in other cancer types have suggested that the poor results testing anti-PD-1 therapy in CRPC may be due to the lack of PD-L1 expression in PCa tumors [7-9]. However, it remains unknown whether patients with ENZ resistant (ENZR) CRPC may be a more relevant cohort to study the efficacy of anti-PD-1 therapies, as expression of PD-L1 on ENZ resistant CRPC and the effects of ENZR tumors on the PD-L1/PD-1 pathway in circulating antigen presenting cells or T cells has not been reported.

In this study, our objective was to determine whether clinically relevant immunotherapy targets, specifically PD-L1/PD-1 and CTLA-4, are upregulated during ENZ resistant CRPC, both in patients and in a pre-clinical model. We show for the first time that ENZ resistance is associated with high frequency of PD-1/L1 therapy targets, not only in the tumor, but in circulating immune cells. Moreover, our pre-clinical results suggest that non-AR driven CRPC phenotypes, such as anaplastic or neuroendocrine cancers, may be especially immunosuppressive.

## RESULTS

### Progression on ENZ in CRPC patients is associated with increased frequency of PD-L1/2^+^ DCs

**Figure 1:**
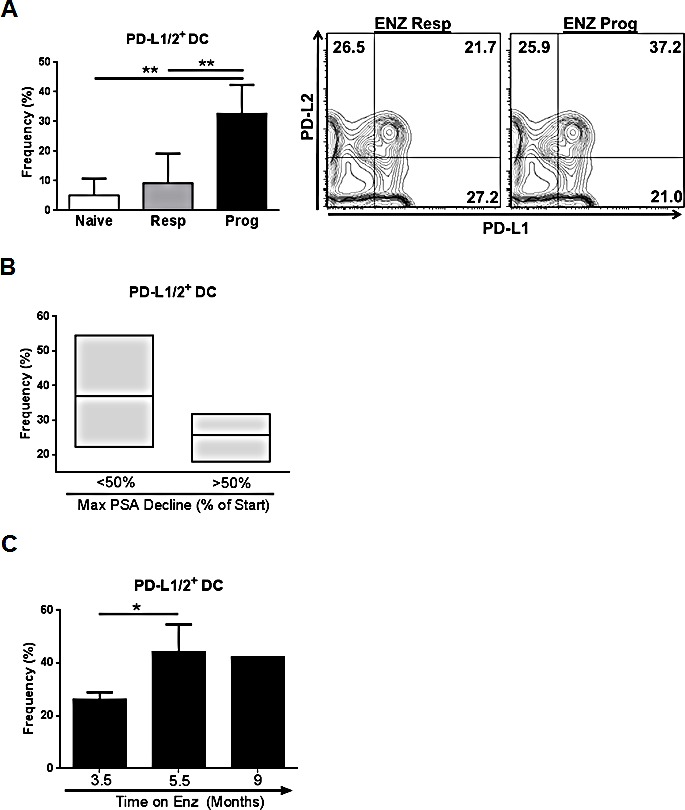
Progression on ENZ in CRPC patients is associated with increased frequency of PD-L1/2 DCs in circulation (A) Evaluation of DCs in blood from CRPC patients: Whole blood was collected from CRPC patients defined as naïve (n=3) responding (resp, n=4) or progressing (prog, n=8) on ENZ at the time of collection and frequency of PD-L1/2^+^ DCs isolated from patient blood was assessed by flow cytometry. Frequency of PD-L1/2^+^ DC (Lin^−^CD11c^+^MHCII^hi^) is shown. Contour plots show DC PD-L1 and PD-L2 expression in representative blood samples, graphs show mean frequency of positive cells +/− SD, ** P=<0.005. (B) Resistance to ENZ in progressing patients is associated with increased PD-L1/2+ DCs: Frequency of PD-L1/2^+^ DCs isolated from blood of progressing patients stratified by maximum PSA decline (% PSA reduction from start of ENZ treatment) is shown. <50% decline, n=5, >50% decline n=3. (C) Time on ENZ increases PD-L1/2+ DC frequency: Frequency of PD-L1/2^+^ DCs isolated from blood of progressing patients stratified by the duration of ENZ treatment is shown. 3.5 mo, n=5; 5.5 mo, n=2; 9 mo, n=1, *P=<0.05. All cell populations are downgated on live, CD45^+^ cells.

Expression of PD-L1/PD-1 in circulating innate immune and T cells is a useful prognostic indicator for aggressive tumor types and Ipilimumab responses [10,11], however no such studies have been reported for CRPC. To determine if PD-L1 pathway targets are increased after ENZ treatment, PD-L1/2 and PD-1 were assessed by flow cytometry on DC and T cells isolated from a small cohort of metastatic CRPC patients who were ENZ naïve or classified as either “progressing” or “responding” to ENZ. We observed a significantly increased frequency of PD-L1/2^+^ DCs in men progressing on ENZ compared to those who responded (p=0.0060), or were naïve (p=.0037), to treatment (Fig.1A). In progressing patients, more PD-L1/2^+^ DCs were associated with poorer response to ENZ treatment and treatment duration. Men who initially responded to ENZ with a <50% decrease in PSA had greater circulating PD-L1/2^+^ DCs than those who had a >50% PSA decline after starting treatment (Fig.1B) and, in progressing patients, PD-L1/2^+^ DC frequency significantly increased with time on ENZ (p=.0497) (Fig.1C). Moreover, in one ENZ progressing patient where serial samples were taken, PD-L1^+^ DC frequency increased after 12 weeks of ENZ (Fig.S1). Examination of checkpoint targets on T cells revealed that although overall frequency of PD-1^+^ CD4^+^ or CD8^+^ T cells was high, no differences in T cell PD-1 expression were observed between patient subsets (Fig.S2A). Comparatively low expression of CTLA-4 on T cells was found across all patients (Fig.S2B). Data from this limited cohort suggests that there is high expression of targetable PD-L/PD-1 pathway molecules in peripheral blood immune cells in patients with ENZ resistant CRPC.

### PD-L1 is upregulated in a pre-clinical model of non-AR driven ENZ resistant CRPC

**Figure 2:**
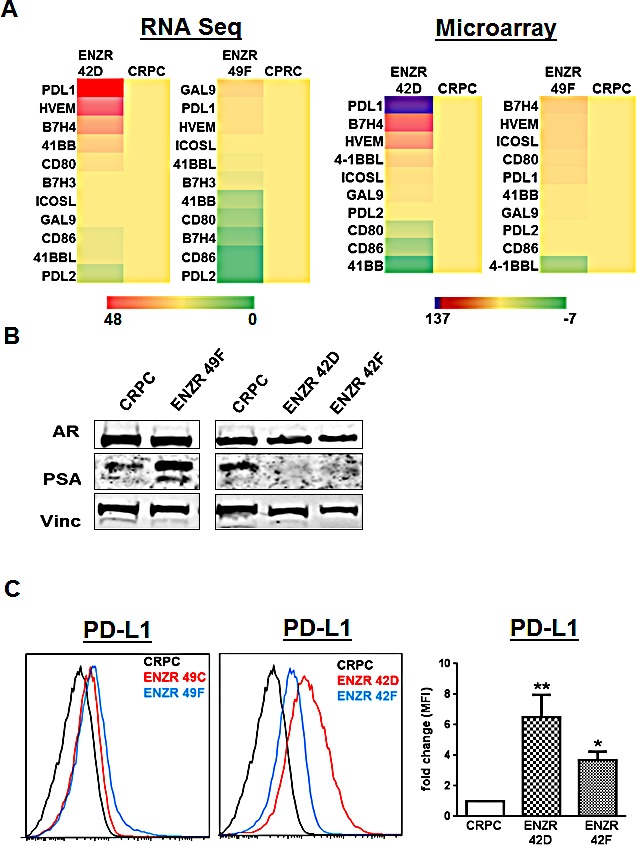
Differential expression of T cell checkpoint molecules in ENZ resistance (A) Expression profile of checkpoint molecules in ENZ resistance: RNA sequencing (left) and microarray (right) data shows average fold change expression in checkpoint molecule genes in ENZ resistant (ENZR) cell lines 42D and 49F compared to CRPC (=1), n=2. (B) Reduced AR activity in ENZR cell lines correlates with PD-L1 expression: CRPC and ENZR cell lines were grown *in vitro* and assessed for AR and PSA expression by western blot, vinculin was used as a loading control. Representative blots from more than three independent experiments are shown. (C) Expression of PD-L1 in ENZ resistant cell lines: Surface expression of PD-L1 on CRPC, ENZR 42D, 42F, 49C and 49F cell lines grown *in vitro* was assessed by flow cytometry and shown as representative histograms from one of three independent experiments, or fold changes in mean fluorescence intensity (MFI) on ENZR 42D and 42F cell lines compared to CRPC (=1). Bar graph shows mean fold MFI changes pooled from three independent experiments, error bars represent SEM, *P=<0.05, ** P=<0.01.

As no matched biopsy specimens were available from our cohort of patients at time of blood collection, we turned to our pre-clinical model to address whether tumor intrinsic PD-L1 expression is associated with ENZ resistance. RNA sequencing of ENZR cell lines showed that PD-L1 was markedly upregulated compared to ENZ sensitive CRPC, and was the most highly expressed B7 family member in the cell line 42D but not in a second ENZR cell line 49F (Fig.2A). The primary distinction between ENZR 42D cells compared to 49F is the activity of the AR; 42D cells express AR but not PSA, whereas 49F cells express both (Fig.2B). Flow cytometry confirmed the significantly increased surface expression of PD-L1 only in two different PSA^−^ ENZR cell lines 42D (p=0.0195) and 42F (p=0.0079) compared to CRPC, and not in the PSA^+^ ENZR cell lines 49C and 49F (Fig.2C). These results suggest that upregulation of immune checkpoint molecules may be one unique mechanism of non-AR driven ENZ resistance.

### Non-AR driven ENZ resistant xenografts increase circulating PD-L1/2^+^ DCs *in vivo*

**Figure 3:**
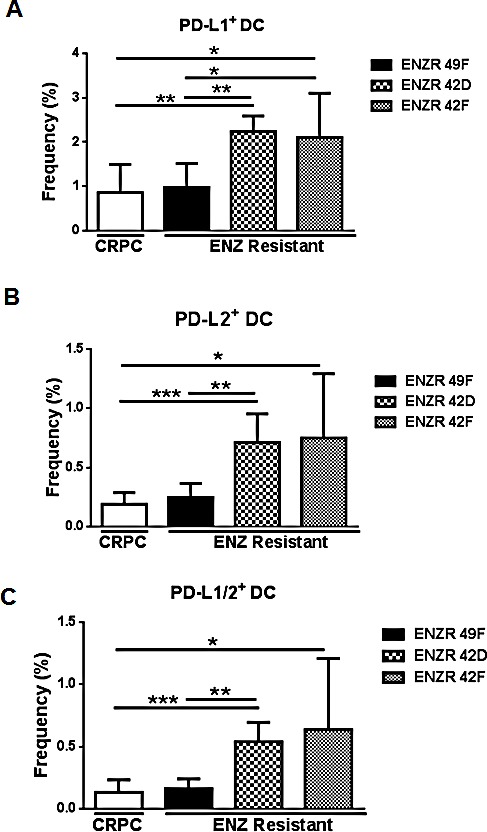
non-AR driven ENZR 42D and 42F xenografts increase circulating PD-L1/2 DCs *in vivo* Evaluation of DCs in blood from mice bearing ENZ resistant tumors: Blood was harvested from mice bearing ENZ resistant (ENZR) or CRPC subcutaneous xenografts when tumors reached 350-650mm^3^ and frequency of PD-L1, PD-L2 and PD-L1/2 double positive DCs isolated from blood was assessed by flow cytometry. Frequency of (A) PD-L1^+^ DC (CD11c^+^MHCII^hi^), (B) PD-L2^+^ DC and (C) PD-L1/2^+^ DC is shown. All cell populations are downgated on live, CD45+ cells. ** P=<0.005, * P=<0.05, ***P=<0.001, error bars on graphs represent SD of representative data from of two independent experiments, n (mouse number)=5-8.

Our patient data suggested that ENZ resistance is associated with increases in PD-L1/2^+^ DCs, and *in vitro* PD-L1 is upregulated on PSA^−^ ENZR cells. Functional suppression of DCs both within the tumor and in the blood occurs in many cancers via upregulation of PD-L1 [12-14], and DC PD-L1 has been linked to tumor intrinsic PD-L1 expression [8]. Accordingly, we found that in tumor-bearing mice, PSA^−^ ENZR 42D and 42F xenografts significantly increased the frequency of PD-L1^+^ (42D p=0.0014, 42F p=0.145), PD-L2^+^ (42D p=0.0004, 42F p=0.0190) and PDL-1/2^+^ DC (42D p=0.0003, 42F p=0.0189) compared to CRPC or to PSA^+^ ENZR 49F xenografts (Fig.3A-C). By contrast, no differences in PD-L1/2^+^ DCs were observed comparing PSA^+^ ENZR 49F to CRPC (Fig.3A-C). These data indicate that PSA^−^ ENZR tumors strongly alter the expression of PD-L1 and PD-L2 on circulating DCs and suggest a link between modulation of tumor intrinsic PD-L1 and DC PD-L1/2 as a mechanism of ENZ resistance specifically when the AR is not classically active.

### Non-AR driven ENZ resistant xenografts prevent PD-L1/2^+^ DC infiltration into tumors

**Figure 4:**
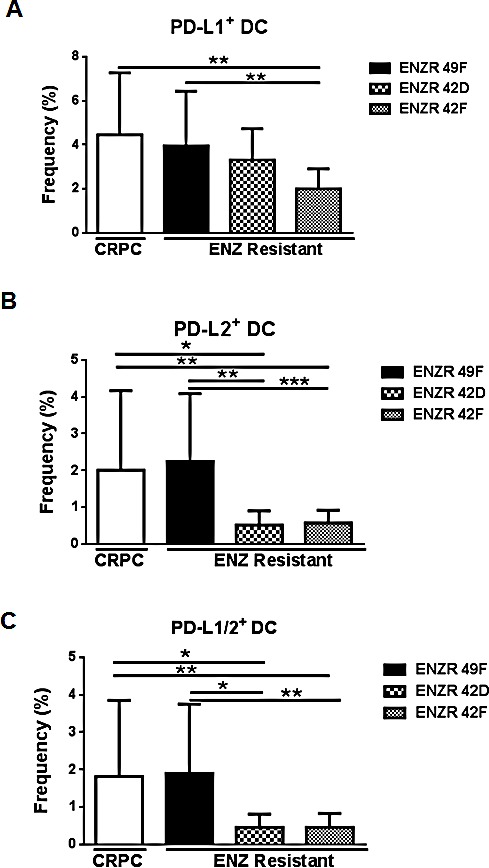
non-AR driven ENZR 42D and 42F xenografts decrease tumor infiltrating PD-L1/2^+^ DCs *in vivo* Evaluation of tumor infiltrating leukocytes: Tumors were harvested from mice bearing ENZ resistant (ENZR) or CRPC subcutaneous xenografts when tumors reached 350-650mm^3^ and frequency of infiltrating PD-L1, PD-L2 and PD-L1/2 double positive DCs isolated from tumors was assessed by flow cytometry. Frequency of (A) PD-L1^+^ DC (CD11c^+^MHCII^hi^), (B) PD-L2^+^ DC and (C) PD-L1/2^+^ DC is shown. All cell populations are downgated on live, CD45^+^ cells. * P=<0.05, ** P=<0.01, ***P=<0.001 error bars on graphs represent SEM of pooled data from two independent experiments, n (tumor number)=11-20.

Depending on tumor type, the presence of tumor infiltrating lymphocytes (TIL) may indicate responsiveness to checkpoint blockade [8]. Contrasting our results in circulating DCs, we found that PSA^−^ ENZR xenografts significantly reduced the frequency of tumor infiltrating PD-L1^+^ (42F p=0.0011), PD-L2^+^ (42D p=0.359, 42F p= 0.0064) and PDL-1/2^+^ DC (42D p=0.0422, 42F p=0.0067) compared to CRPC or to PSA^+^ ENZR 49F xenografts (Fig.4A-C). Similar to our previous results, PSA^+^ ENZR 49F tumors did not prevent infiltration of PD-L expressing DCs compared to CRPC (Fig.4A-C). Although the modulation of PD-L1/2^+^ DC populations differs between the circulation and tumor itself, our results suggest that in both locations, PSA^−^ ENZR xenografts modulate DC PD-L1/2 expression more than CRPC or PSA^+^ ENZR cells, underscoring the potentially immunosuppressive features of non-AR driven resistant disease.

## DISCUSSION

The efficacy of immune checkpoint blockade immunotherapies like Ipilimumab and PD-1 pathway inhibitors in CRPC patients remains questionable. Despite one CRPC patient showing a complete response to Ipilimumab [15], in another study there was no improvement in overall survival for CRPC patients [16] and no objective responses were observed in 17 CRPC patients treated with anti-PD-L1 antibody [7]. Moreover, the positive correlation between tumor PD-L1 expression and response to PD-1 pathway immunotherapies [8] and the fact that PD-L1 expression on CRPC tumors has been hard to identify [7,8], has made justifying the use of PD-1 blockade even more difficult for CRPC patients. However, the fact that not all patients who respond to PD-1 or PD-L1 therapies exhibit tumor expression of PD-L1, and that only a very small sample of CRPC tumors have been assessed for PD-L1 expression [7], suggests a re-examination of the criteria that could define responsiveness to checkpoint blockade therapies in CRPC patients is required.

In particular, it is essential to assess which immune evasion strategies are employed by ENZ resistant tumors that utilize AR re-activation as a main driver of resistance vs. those that do not. Non-AR driven CRPC is clinically relevant, as it is has been estimated that up to 25% of men that die from advanced CRPC have a disease not driven by the AR [17]. With the increasing use of potent anti-androgens in the clinic that limit AR activity but not expression, such as ENZ, there is increasing concern that more patients may present with a non-AR driven phenotype of disease. Indeed, most cases of neuroendocrine prostate cancer, or anaplastic prostate cancer arise after hormone therapy [18] and the evolution of an AR-neuroendocrine phenotype from prostate adenocarcinoma is a proposed mechanism of anti-androgen resistance [19,20]. Recently, a number of tumor intrinsic ENZ resistance mechanisms dependent on the AR have been identified in metastatic CRPC [21], but differences in immune responses in these patients were outside the scope of this work. Thus, ours are the first study to indicate that expression of PD-L1 on tumor cells may be a unique mechanism of ENZ resistance that is independent of AR re-activation and not observed in CRPC. This is in line with reports showing that CRPC does not express high levels of PD-L1 [7,8]. As tumor cell expression of PD-L1 is an important prognostic indicator for tumor regression with anti-PD-1 therapy in other cancers [8], our results highlight the importance of investigating the functional ramifications of PD-L1 expression by ENZR tumors as well as tumor expression of PD-L1 expression in patients on ENZ, particularly in men with disease that is non-AR driven.

Our results also suggest that ENZ resistant CRPC may suppress immune responses not only via tumor intrinsic PD-L1 expression, but also through the induction of PDL-1/2 and/or PD-1 on circulating innate immune cells. We show for the first time that patients progressing on ENZ have significantly higher frequency of PD-L1/2^+^DCs in circulation, which increases with time on ENZ and was associated with a poorer initial response to ENZ treatment. These patient data support our pre-clinical findings, which showed that ENZR xenografts could cause significant increases in PDL1/2^+^ DCs in the blood of tumor bearing mice. Like tumor intrinsic PD-L1 expression, this was a feature unique to non-AR driven tumors, which are particularly aggressive in patients [18]. These results are in accordance with various studies showing that increased DC PD-L1/2 expression correlates with poor outcome of aggressive tumors [14] such as glioblastoma [13] and pancreatic cancer [12].

Although not able to assess T cell populations in our pre-clinical model, we did find frequencies of PD-1^+^ CD4^+^ and CD8^+^ T cells were high in ENZ progressing patients, however they were similar to patients naïve to or responding to treatment. These results suggest that DCs may be a cell population more indicative of changes in PD-L1 pathway activity as CRPC progresses on ENZ treatment. However, these results also may be indicative of the phenotype of T cells in the tumor microenvironment as well. Although no matched biopsy specimens were available for our patient cohort, the high frequencies of circulating PD-1^+^T cells may be suggestive of high levels of infiltrating PD-1^+^ cells in tumors, as this correlation has been shown in patients with high Gleason grade prostate cancer as well as renal cancer [22,23]. Since tumor PD-1^+^T cells are associated with poor prognosis in both renal cell [24] and hepatocellular carcinoma [25], our data showing high levels of PD-1^+^T cells in advanced CRPC patients may have important clinical implications.

By contrast to PD-1^+^ T cell frequencies, we found that all patients showed relatively low frequencies of CTLA-4^+^ T cell subsets. While this observation could suggest PD-L1 is a more dominant checkpoint pathway that is activated during ENZR CRPC, it is important to highlight that responses to Ipilimumab in prostate cancer patients does not always correlate with high frequency of CTLA-4^+^T cells [26] and it is unknown whether expression of CTLA-4 ligands CD80/86 on antigen presenting cells correlate with positive or negative responses to CTLA-4 blockade. In addition, despite lack of immune correlates to suggest an activation of the CTLA-4 pathway, in one recent publication, a patient with metastatic CRPC showed complete responses to Ipilimumab [15]. Overall these data highlight that there may be subsets of CRPC patients that will respond to either PD-1 or CTLA-4 immunotherapies, and our data suggest further investigation into circulating cells as immune correlates of responses may be useful in predicting response. Indeed, recent evidence suggests that increased overall survival of prostate cancer patients treated with Ipilimumab and the vaccine GVAX was associated with increased with pre-treatment levels of CTLA-4^+^ and PD-1^+^ T cells in circulation [27]. Although these data contrast an investigation into immune correlates in PROSTVAC-Ipilimumab treated patients [26], both data sets support the relevance of surveying peripheral immune responses in advanced prostate cancer patients to predict immunotherapy outcome.

Finally, our results suggest a potential third mechanism for immune evasion during ENZ resistance, through limiting DC infiltration into the tumor. Contrasting our results in the blood of tumor bearing mice, we found that infiltration of PD-L1/2^+^ DCs was limited by non-AR driven ENZR tumors. These results suggest that PSA^−^ ENZR tumors may prevent innate cell activation and infiltration in the immediate tumor microenvironment while suppressing the activity of mature DCs in the periphery. The relevance of TIL populations to immunotherapy outcomes remains unclear, as this indicator seems to be dependent on tumor type. For example, in melanoma, TIL infiltration is a good prognostic indicator for response to Ipilimumab whereas no significant relationship has been shown between TIL infiltration and response to anti-PD-1 therapy in renal, lung and colorectal cancer [8]. Importantly however, these IHC studies have assessed both T cell and innate cell populations, which most likely play distinct roles in dictating anti-tumor responses during immunotherapy. Indeed, data showing that prostate cancer patients with high Gleason score tumors show a strong correlation between peripheral blood and tumor infiltrating PD-1^+^ T cells [23], suggests that the peripheral response still may be an easily accessible indicator for the activity of the PD-L1/PD-1 pathway. Given the difficulty of obtaining tumor tissue from metastases from CRPC patients, exploring minimally invasive approaches for interrogating potential circulating biomarkers (PD-L1/2^+^ DCs and PD-1^+^ T cells) and how they correlate to tumor PD-L1 expression is especially attractive.

Taken together, our data suggest that ENZR CRPC in mouse models and patients is associated with strong expression of the targets for anti-PD-1 therapy. Moreover, our pre-clinical data underscores the potentially disparate immunomodulatory effects of AR-driven vs. non-driven ENZR tumors, which may add to establishing a predictive signature of resistance to ENZ [28] or stratify patient subsets most amenable to checkpoint blockade. The clinical relevance of this observation should be more thoroughly investigated, and future studies that examine the utility of monitoring circulating cell PD-L1 pathway activity vs. tumor intrinsic PD-L1 expression in CRPC patients to predict responsiveness to checkpoint blockade immunotherapy are warranted.

## METHODS

### Patients

Whole blood was collected for peripheral blood mononuclear cell (PBMC) isolation from metastatic castration-resistant prostate cancer (CRPC) patients (median age=73 years, range 61-88) prior to (naïve, n=3) or after receiving 160 mg PO (by mouth) Enzalutamide (ENZ) daily for a minimum of 12 weeks. At time of blood collection, ENZ treated patients were classified as “responding” or “progressing”. Responding patients (n=4) had prostate specific antigen (PSA) decline ≥ 50% from baseline with no evidence of biochemical or radiographic progression (Prostate Cancer Working Group 2 criteria, PCWG2 [29]), or clinical progression. Clinical progression was defined as worsening of disease-related symptoms necessitating change in anti-neoplastic therapy and/or decrease in Eastern Cooperate Oncology Group (ECOG) Performance status ≥ 2 levels [30]. Progressing patients (n=8) had evidence of biochemical and/or radiographic (PCWG2 criteria) and/or clinical progression.

### Cell Culture and Western Blotting

Enzalutamide (ENZ) resistant (ENZR) and ENZ sensitive CRPC cell lines were generated from an *in vivo* LNCaP model of CRPC; CRPC cells were derived from vehicle treated LNCaP tumors that recurred as CRPC after castration and treated with vehicle control, while ENZR cells were derived from CRPC tumors treated with ENZ that recurred [2]. Cell lines derived from ENZR xenografts were given numerical and alphabetical designations corresponding to individual tumors and mice from which they were derived (ENZR 42D, 42F or 49F). Cell lines were tested and authenticated by whole-genome and whole-transcriptome sequencing (Illumina Genome Analyzer IIx, 2012). Cells were maintained in RPMI-1640, 10% fetal bovine serum (FBS), 100 U/mL penicillin-G, 100 mg/mL streptomycin (Gibco), +10uM ENZ or DMSO vehicle. For flow cytometry, RNA or protein isolation, cells were seeded at a density of 1M cells/10mls media and harvested after 72 hours. AR and PSA levels were assessed by standard SDS-PAGE and western blotting using anti-androgen receptor (AR) and PSA antibodies (Santa Cruz Biotechnology) as previously described [3].

### RNA Sequencing/Microarray

RNA-seq was performed cells using Illumina HiSeq 2000 at BGI according to standard protocols. Sequence data mapping and processing was performed as previously described, except normalization was performed using reads per million [19]. Microarray gene expression was performed as previously described [19] using Agilent SurePrint G3 Human GE 8×60K slides (Design ID 028004) and analyzed using Agilent GeneSpring 11.5.1 and Ingenuity Knowledge Base (Ingenuity Systems). Two experimental replicates of ENZR and CRPC cells were used.

### Xenograft Studies

CRPC and ENZR tumors were grown and monitored in castrated male athymic mice (Harlan Sprague-Dawley, Inc) in the presence or absence of ENZ as previously described [2,3]. When tumors reached 350mm^2^ to 650mm^2^, blood and tumors were harvested for flow cytometric analysis. All animal procedures were conducted according to the guidelines of the Canadian Council on Animal Care.

### Flow Cytometry

Cells were removed from plates using 1ml of 1× Citric Saline for 10min at room temp and washed 1× in RPMI+10%FBS. Cells from murine whole blood and tumors were isolated as previously described [31]. Human PBMCs from whole blood were isolated using Ficoll Paque Plus (GE Healthcare) according to manufacturer's instructions. Before antibody addition, cells were incubated with either mouse Fc block (2.4G2) or Human Fc Receptor Binding Inhibitor (eBioscience) for 20min on ice. Flow cytometry staining was performed using anti-human PD-L1, PD-L2, CD11c (eBioscience), lineage cocktail (CD3, CD14, CD19, CD20, and CD56-Biolegend) or anti-mouse CD11c, PD-L1 (Biolegend), PD-L2 (eBioscience) as described [31] followed by staining with Fixable Viability Dye eFluor 506 (eBioscience, per instructions) and fixation in 2% paraformaldehyde (PFA). Data were acquired (minimum 10K events) on a Canto II (BD Biosciences) and analyzed with FlowJo (TreeStar).

### Statistical Analysis

Unpaired, two-tailed, student's T tests were performed to analyze statistical significance between frequencies or mean fluorescence intensities of assessed cell populations using Graph Pad Prism (Graph Pad Software).

## SUPPLEMENTARY MATERIALS AND FIGURES


