# Supplementary material for: PD-L1 is highly expressed in Enzalutamide resistant prostate cancer
Source: Oncotarget. 2014 Nov 6;6(1):234–42. doi: 10.18632/oncotarget.2703 (PMC4381591; doi:10.18632/oncotarget.2703)
Supplement: Supplementary file 1 [file oncotarget-06-234-s001.pdf]

PD-L1 is highly expressed in Enzalutamide resistant prostate cancer

Supplementary Material

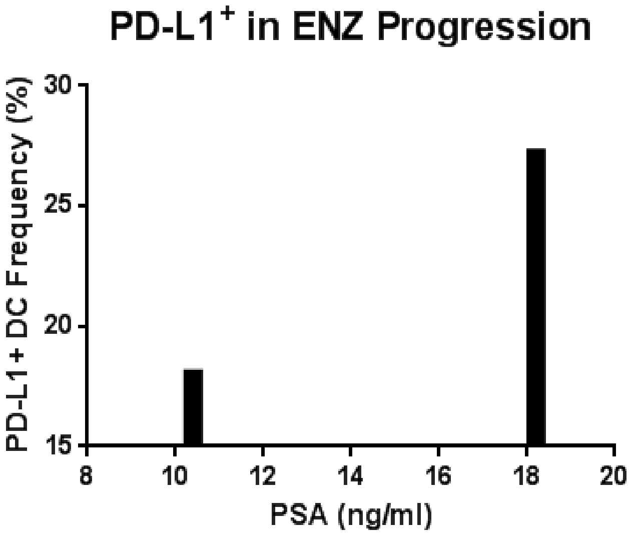

Supplementary Figure 1: Frequency of circulating PD-L1$^+$ DCs increases after progression on ENZ. Evaluation of DCs from a serially sampled CRPC patient: Whole blood was collected from the same CRPC patient prior to ENZ treatment (naïve, PSA=10.4ng/ml) or after 12 weeks on ENZ treatment at which he was defined as progressing (PSA=18.2 ng/ml) and frequency of PD-L1$^+$ DCs isolated from patient blood was assessed by flow cytometry. Frequency of PD-L1$^+$ DC (Lin$^-$CD11c$^+$MHCII^{hi}) is shown vs. circulating PSA value. All cell populations are downgated on live, CD45$^+$ cells.

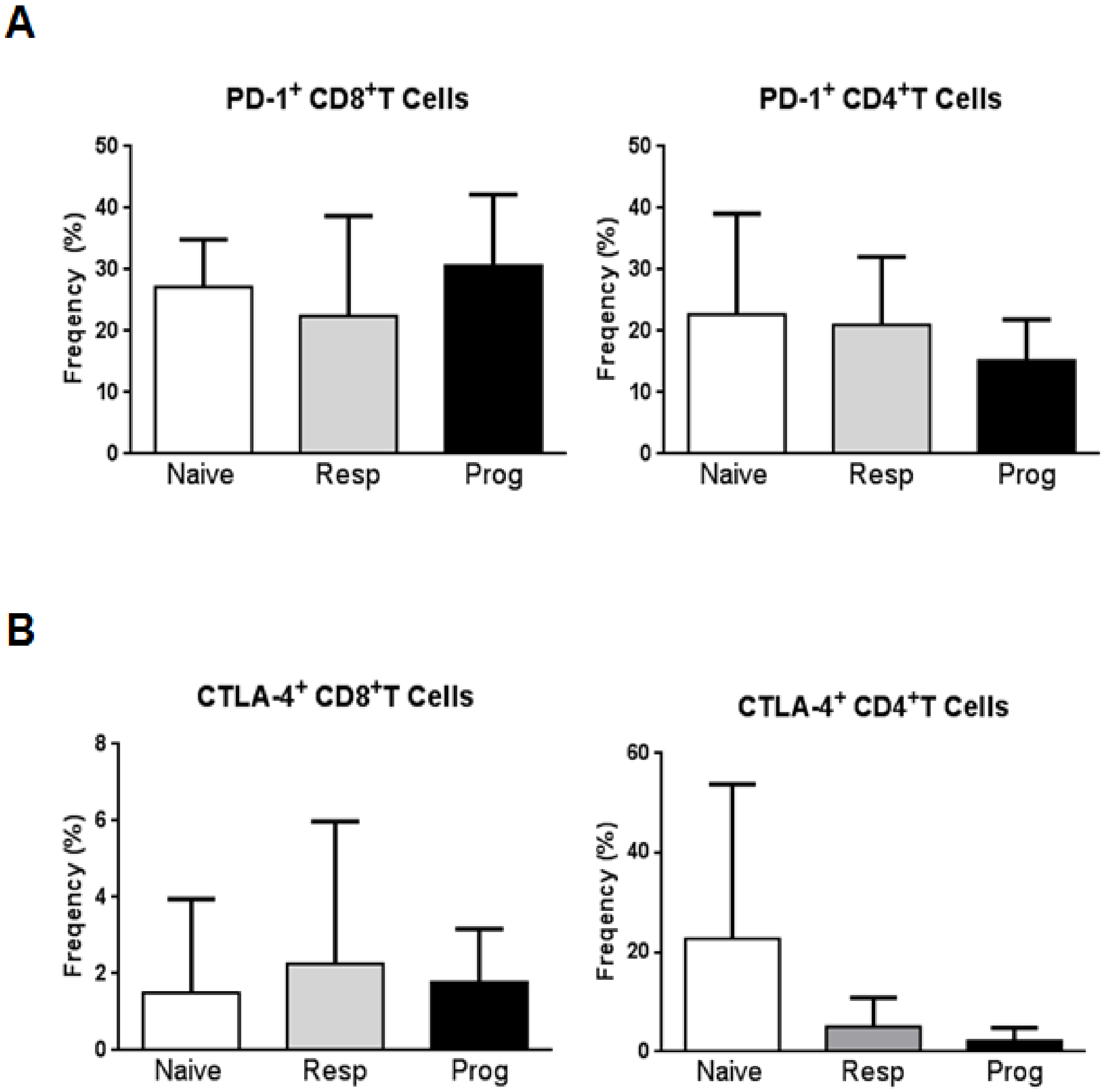

Supplementary Figure 2: Frequency of circulating PD-1$^+$ and CTLA-4$^+$ T cells in CRPC patients progressing on ENZ. Evaluation of T cells in blood from CRPC patients: Whole blood was collected from CRPC patients defined as naïve (n=3) responding (resp, n=4) or progressing (prog, n=8) on ENZ at the time of collection and frequency of PD-1$^+$ and CTLA-4$^+$ T cells isolated from patient blood was assessed by flow cytometry. Frequency of **(A)** PD-1$^+$ or **(B)** CTLA-4$^+$ T cells (CD3$^+$CD8$^+$ or CD3$^+$CD4$^+$) is shown. Mean frequency of positive cells +/- SD is shown. All cell populations are downgated on live, CD45$^+$ cells.
